# *In Utero* Pesticide Exposure and Leukemia in Brazilian Children < 2 Years of Age

**DOI:** 10.1289/ehp.1103942

**Published:** 2012-10-22

**Authors:** Jeniffer Dantas Ferreira, Arnaldo Cézar Couto, Maria S. Pombo-de-Oliveira, Sergio Koifman, the Brazilian Collaborative Study Group of Infant Acute Leukemia

**Affiliations:** 1 Environment and Public Health Post-graduation Program, National School of Public Health, Oswaldo Cruz Foundation (FIOCRUZ), Rio de Janeiro, Brazil; 2 Pediatric Hematology–Oncology Program, Research Center, Instituto Nacional de Câncer/Rio de Janeiro, Rio de Janeiro, Brazil

**Keywords:** childhood cancer, infant leukemia, lactation, pesticides, pregnancy

## Abstract

Background: An association between pesticide exposure and cancer has been suggested. Infant leukemia is a rare neoplasm and its association with maternal pesticide exposure has been poorly explored.

Objectives: We investigated the association between pesticide exposure during pregnancy and leukemia in children < 2 years of age.

Methods: A hospital-based case–control study was carried out in 13 Brazilian states during 1999–2007. Mothers of 252 cases and those of 423 controls were interviewed. Information on pesticide exposures 3 months before pregnancy, throughout pregnancy, and during breastfeeding was obtained. Unconditional logistic regression was used to estimate adjusted odds ratios (aORs) for associations between pesticide exposures and leukemia.

Results: Associations with ever use of pesticides during pregnancy were observed for acute lymphoid leukemia (ALL) (aOR = 2.10; 95% CI: 1.14, 3.86) and acute myeloid leukemia (AML) (aOR = 5.01; 95% CI: 1.97, 12.7) in children 0–11 months of age, and with ALL (aOR = 1.88; 95% CI: 1.05, 5.23) at 12–23 months of age. According to reported maternal exposure to permethrin, higher risk estimates were verified for children 0–11 months of age (aOR = 2.47; 95% CI: 1.17, 5.25 for ALL; and aOR = 7.28; 95% CI: 2.60, 20.38 for AML). Maternal pesticide exposure related to agricultural activities showed an aOR of 5.25 (95% CI: 1.83, 15.08) for ALL, and an aOR of 7.56 (95% CI: 1.83, 31.23) for AML.

Conclusions: These results support the hypothesis that pesticide exposure during pregnancy may be involved in the etiology of acute leukemia in children < 2 years of age.

Pesticide exposure is a public health concern worldwide. In Brazil, a study conducted in two stages among small-scale fruit farmers revealed that 19% of them had reported at least one poisoning episode (4% in the year before interview). Even at this stage, 11% of all episodes were probable cases of acute poisoning according to World Health Organization criteria (Faria et al. 2009). Moreover, pesticide use also has been associated with chronic diseases such as cancer, including childhood leukemia, at 0–14 years of age (Bassil et al. 2007; Infante-Rivard and Weichenthal 2007; Turner et al. 2010; Zahm and Ward 1998).

Infant leukemias are rare acute leukemias diagnosed within the first 12 months of age, although some researchers include cases up to 18 months of age (Alexander et al. 2001; Pombo-de-Oliveira and Koifman 2006). DNA alterations during the periconceptional period or pregnancy, which may be caused by chemical substances and other environmental exposures, are potential risk factors for infant leukemias (Buffler et al. 2005; Lafiura et al. 2007). Previous studies have reported associations between childhood leukemia and maternal exposure to pesticides (Ma et al. 2002; Meinert et al. 2000; Menegaux et al. 2006; Monge et al. 2007; Rudant et al. 2007).

However, few studies of pesticides and leukemia in very young children have been published. Additionally, most studies of association between childhood leukemias and pesticides have focused on parents’ occupational exposures (Colt and Blair 1998; Monge et al. 2005; Perez-Saldivar et al. 2008; Rudant et al. 2007; Wigle et al. 2009), and a few have considered the use of household pesticides during the prenatal period (Lafiura et al. 2007; Meinert et al. 2000; Monge et al. 2007; Zahm and Ward 1998).

A case–control study of risk factors for leukemia in children < 2 years of age was conducted in Brazil, and an adjusted odds ratio (aOR) of 2.18 (95% CI: 1.53, 2.13) was reported in association with maternal exposure to pesticides (Pombo-de-Oliveira and Koifman 2006). In the present investigation, we aimed to extend these analyses of maternal pesticide exposure and leukemia in the offspring.

## Methods

*Study population*. This investigation is part of a multicenter study, the Multi-institutional Study of Infant Leukemia: Contribution of Immunomolecular Markers in Distinguishing Different Etiopathogenic Factors, which focuses on the investigation of biomarkers of leukemia diagnosed in children < 2 years of age in Brazil. Participants (*n* = 675) were recruited from 13 states in all geographic areas in the country but the Amazon, including cities in the Southern Region, the Southeast, the Northeast, and the Middle West.

*Study design*. This is a hospital-based multicenter case–control study in which controls were frequency matched with leukemia cases according to age (0–23 months) and enrolled from the same geographic areas where cases were diagnosed.

Data were obtained by in-person interviews carried out from 1999 through 2007 with mothers of newly diagnosed patients. These patients were recruited from the Brazilian National Health System centers that provide free oncologic care for pediatric patients and from general hospitals.

Cases (*n* = 252) were defined as children < 24 months of age with a conclusive diagnosis of acute lymphoid leukemia (ALL) (*n* = 193) or acute myeloid leukemia (AML) (*n* = 59) confirmed by morphology, immunophenotype, and standard cytogenetic-molecular methods.

Controls (*n* = 423) were selected from among children < 24 months of age with nonmalignant diseases who were patients at the Brazilian National Health System centers where the cases were recruited or patients of general hospitals in the same cities. The hospitals from which controls were recruited had the same catchment areas of those of cases. Controls included children with infectious and parasitic diseases (*n* = 124, 29.4%), nonmalignant hematological diseases (*n* = 83, 19.6%), asthma and bronchitis (*n* = 43, 10.2%), hemangioma (*n* = 40, 9.4%), severe diarrhea (*n* = 39, 9.2%), cardiovascular diseases (*n* = 25, 5.8%), and other nonmalignant conditions (*n* = 69, 16.4%).

Children with congenital syndromes, myelodisplasia, adoptive parents, or unknown biological mothers were not eligible to be enrolled, and controls with a cancer diagnosis were excluded. Participation of invited cases and controls in the study was, respectively, 96% and 95% (Pombo-de-Oliveira and Koifman 2006).

*Data collection*. The study was specifically designed to collect information on several environmental exposures potentially associated with leukemogenesis. Data were collected by in-person interviews with case and control mothers at the hospital. A standardized questionnaire used for all participants contained information on environmental exposures during pregnancy, including use of pesticides. Interviewers were health staff members without special knowledge of pesticide toxicology, and were instructed to register all answers provided by the interviewed mothers. Pesticide exposure information was further analyzed and classified according to toxicological characteristics. Child skin color was indicated by mothers and further dichotomized as white and nonwhite. At each center, the same interviewers were responsible for both cases and controls. Participating mothers provided written informed consent for themselves and their children.

Pesticide exposure was evaluated based on the mother’s report of any contact with pesticides (at least once) during the 3 months before pregnancy (periconceptual period), throughout each pregnancy trimester, or during the 3 months after birth (breastfeeding). They were requested to inform about any contact with pesticides at home or in the workplace during each of these pregnancy times windows of exposure. Brand names of commercial products reported by the mothers were used to determine chemical content, and associations with pesticides were explored according to the form of use—unintentional, domestic (household), or agricultural (maternal occupational exposure, or living in an agricultural area with pesticides use)—and duration and regularity of contact (no use, once a week or less, more than once a week).

Exposures to products that included pesticides from multiple chemical classes (for instance, pyrethroids, organophosphates, and carbamates), or maternal exposure to both insecticides and herbicides were also assessed, and categorized as mixed exposures whenever reported.

*Ethics.* This investigation was approved by the Research Ethics Committee of the Brazilian National Cancer Institute (No. 005/06) and by the Research Ethics Committee of the Oswaldo Cruz Foundation (FIOCRUZ), No. 32/10. Participating mothers provided written informed consent for themselves and their children.

*Statistical analysis*. We performed unconditional logistic regression to estimate associations between pesticide exposures and early leukemias ORs and their 95% CIs after adjustment for birth weight (< 4,000 g, ≥ 4,000 g), maternal age at birth (< 35 years, ≥ 35 years), maternal schooling (≤ 8 years, > 8 years), oral contraceptive intake during pregnancy (no use, use during pregnancy), and child’s skin color (white or nonwhite).

We performed sensitivity analysis using different control subsets, specifically *a*) after excluding controls with gastro-intestinal infections, parasitic diseases, dehydration, malnutrition or diarrhea; *b*) controls with respiratory illnesses, including tuberculosis, pneumonia, asthma, bronchitis, and bronchiolitis. Considering that both subsets are more prevalent among low-income strata, such procedures aimed to evaluate possible confounding resulting from these controls inclusion. In addition, we estimated stratum-specific ORs according to child’s skin color.

## Results

Geographical distribution of participants is presented in Supplemental Material, Table S1 (http://dx.doi.org/10.1289/ehp.1103942), showing a higher number of children from São Paulo and Rio de Janeiro. Sociodemographic characteristics differed between cases and controls. Specifically, cases were more likely than controls to be white, to have mothers who were older at child’s birth and had > 8 years of education, and to have higher family income (Table 1). Complete information on model covariates was available for 85% of cases and 90% of controls. Agricultural pesticide exposures were reported by 22 (3.3%) mothers, including 7 (1.0%) who were agricultural workers.

**Table 1:** Distribution [n (%)] of selected sociodemographic variables; leukemia cases and controls, children < 2 years of age, Brazil, 1999–2007.

Characteristic	Cases (n = 252)	Controls (n = 423)	p-Value
Sex
Male	130 (51.6)	226 (53.4)	0.643
Female	122 (48.4)	197 (46.6)
Missing	0	0
Birth weight
< 4,000 g	234 (92.9)	393 (93.0)	0.470
≥ 4,000 g	16 (6.3)	21 (5.0)
Missing	2 (0.8)	9 (2.0)
Child’s skin color
White	170 (67.5)	153 (36.2)	< 0.001
Nonwhite	77 (30.5)	256 (60.5)
Missing	5 (2.0)	14 (3.3)
Place of birth
Northeast	52 (20.6)	101 (24.1)	0.552
Midwest	18 (7.1)	31 (7.3)
Southeast	155 (61.5)	237 (56.0)
South	27 (10.7)	54 (12.6)
Missing	0	0
Maternal age (years)a
< 18	8 (3.2)	60 (14.1)	< 0.001
18–24	91 (36.1)	182 (42.9)
25–34	117 (46.4)	145 (34.2)
> 35	36 (14.3)	36 (8.8)
Missing	0	0
Maternal education (years)
≤ 8	81 (32.1)	206 (48.6)	< 0.001
> 8	146 (57.9)	209 (49.4)
Missing	25 (10.0)	8 (2.0)
Family income (Brazilian real)
≤ 350	74 (29.4)	148 (35.0)	< 0.001
351–1,750	102 (40.5)	218 (51.5)
1,751–3,500	27 (10.7)	11 (2.6)
≥ 3,500	12 (5.0)	3 (0.7)
Missing	37 (14.4)	43 (10.2)
Oral contraceptive use
No	223 (88.5)	404 (95.5)	< 0.001
Yes	29 (11.5)	19 (4.5)
Missing	0	0
^*a*^Maternal age at birth.			

Pesticide use at any time during the pregnancy was reported by 60.7% of AML case mothers, 36.4% of ALL case mothers, and 21.3% of control mothers of children who were 0–11 months of age (Table 2). Adjusted odds ratios (aORs) were 2.10 (95% CI: 1.14, 3.86) for ALL and 5.01 (95% CI: 1.97, 12.7) for AML.

**Table 2:** Maternal exposure to pesticides by time window of exposure; leukemia cases and controls, children < 2 years of age, Brazil, 1999–2007.

Pesticide exposure	Controls (n = 423) [n (%)]	ALL (n = 193) [n (%)]	AML (n = 59) [n (%)]	ALL		AML	
				Crude OR (95% CI)	aORa (95% CI)	Crude OR (95% CI)	aORa (95% CI)
Pesticide use
0–11 months
No	200 (78.7)	56 (63.6)	11 (39.3)	1.00	1.00	1.00	1.00
Yes	54 (21.3)	32 (36.4)	17 (60.7)	2.12 (1.25–3.59)	2.10 (1.14–3.86)	5.72 (2.53–12.94)	5.01 (1.97–12.75)
Missing	0	0	0
12–23 months
No	116 (68.6)	55 (52.4)	16 (51.6)	1.00	1.00	1.00	1.00
Yes	53 (31.4)	50 (47.6)	15 (48.4)	1.99 (1.20–3.29)	1.88 (1.05–5.23)	2.05 (0.95–4.46)	1.98 (0.83–4.74)
Missing	0	0	0
Periconceptualb
0–11 months
No	220 (86.6)	63 (71.6)	15 (53.6)	1.00	1.00	1.00	1.00
Yes	32 (12.6)	18 (20.4)	8 (28.6)	1.96 (1.03–3.73)	2.40 (1.20–4.81)	3.67 (1.44–9.34)	3.81 (1.34–10.84)
Missing	2 (0.8)	7 (8.0)	5 (17.8)
12–23 months
No	125 (74.0)	64 (61.0)	15 (48.4)	1.00	1.00	1.00	1.00
Yes	36 (21.3)	32 (30.5)	7 (22.6)	1.62 (0.61–4.28)	1.34 (0.47–3.85)	1.94 (1.04–3.61)	2.48 (1.20–5.11)
Missing	8 (4.7)	9 (8.5)	9 (29.0)
1st Trimester
0–11 months
No	219 (86.2)	63 (71.6)	16 (57.2)	1.00	1.00	1.00	1.00
Yes	32 (12.6)	18 (20.4)	7 (25.0)	1.96 (1.03–3.72)	1.86 (0.94–3.72)	2.99 (1.14–7.84)	2.75 (0.96–7.92)
Missing	3 (1.2)	7 (8.0)	5 (17.8)
12–23 months
No	127 (75.1)	65 (61.9)	20 (64.5)	1.00	1.00	1.00	1.00
Yes	35 (25.0)	31 (29.5)	8 (25.8)	1.73 (0.98–3.05)	1.87 (0.99–3.56)	1.45 (0.59–3.57)	1.28 (0.47–3.53)
Missing	7 (4.1)	9 (8.5)	3 (9.7)
2nd Trimester
0–11 months
No	219 (86.2)	64 (72.7)	16 (57.2)	1.00	1.00	1.00	1.00
Yes	32 (12.6)	17 (19.3)	7 (25.0)	1.70 (0.89–3.25)	1.75 (0.87–3.55)	2.80 (1.08–7.32)	2.27 (0.79–6.47)
Missing	3 (1.2)	7 (8.0)	5 (17.8)
12–23 months
No	129 (76.2)	68 (64.8)	20 (64.5)	1.00	1.00	1.00	1.00
Yes	33 (19.5)	28 (26.7)	8 (25.8)	1.61 (0.90–2.88)	1.76 (0.91–3.39)	1.56 (0.63–3.86)	1.48 (0.54–4.04)
Missing	7 (4.1)	9 (8.5)	3 (9.7)
3rd Trimester
0–11 months
No	218 (85.8)	65 (73.8)	15 (53.6)	1.00	1.00	1.00	1.00
Yes	33 (13.0)	16 (18.2)	8 (28.6)	1.63 (0.84–3.14)	1.88 (0.93–3.79)	3.52 (1.39–8.96)	3.70 (1.32–10.38)
Missing	3 (1.2)	7 (8.0)	5 (17.8)
12–23 months
No	123 (72.8)	68 (64.8)	20 (64.5)	1.00	1.00	1.00	1.00
Yes	39 (23.1)	28 (26.7)	8 (25.8)	1.30 (0.74–2.29)	1.26 (0.66–2.40)	1.26 (0.52–3.09)	0.97 (0.35–2.69)
Missing	7 (4.1)	9 (8.5)	3 (9.7)
Breastfeedingc
0–11 months
No	229 (90.2)	69 (78.4)	14 (50.0)	1.00	1.00	1.00	1.00
Yes	22 (8.6)	12 (13.6)	9 (32.2)	1.81 (0.85–3.84)	2.05 (0.92–4.58)	6.69 (2.60–17.21)	7.04 (2.47–20.10)
Missing	3 (1.2)	7 (8.0)	5 (17.8)
12–23 months
No	128 (75.7)	68 (64.8)	21 (67.7)	1.00	1.00	1.00	1.00
Yes	33 (19.6)	28 (26.7)	7 (22.6)	1.60 (0.89–2.86)	1.53 (0.80–2.95)	1.29 (0.51–3.30)	1.20 (0.43–3.34)
Missing	8 (4.7)	9 (8.5)	3 (9.7)
aaORs by use of oral contraceptives during pregnancy, maternal age and education, child’s birth weight and skin color. bThree months before pregnancy. cThree months after delivery.								

For children diagnosed or enrolled at 12–23 months of age, 48.4%, 47.6%, and 31.4% of AML, ALL, and controls were exposed to pesticides during pregnancy, respectively. Adjusted ORs were 1.88 (95% CI: 1.05, 5.23) for ALL and 1.98 (95% CI: 0.83, 4.74) for AML.

Among children diagnosed or enrolled at 0–11 months of age, information on periconceptual pesticide exposure was available for 99% of controls, 92% of ALL cases, and 82% of AML cases. At 12–23 months of age, information was available for, respectively, 95%, 63%, and 71% (Table 2). ALL and AML were significantly associated with periconceptual exposures among children 0–11 months of age (aOR 2.40; 95% CI: 1.20, 4.81; and aOR 3.81; 95% CI: 1.34, 10.8, respectively). AML was significantly associated with periconceptual exposure in children 12–23 months of age (aOR 2.48; 95% CI: 1.20, 5.11).

The odds of AML were increased with exposure during all time periods among children 0–11 months of age, with significant associations for pesticide exposure in the third trimester (aOR 3.70; 95% CI: 1.32, 10.4) and during breastfeeding (aOR 7.04; 95% CI: 2.47, 20.1). At 12–23 months of age, the odds were, respectively, aOR 0.97; 95% CI: 0.35, 2.69 and aOR 1.20; 95% CI: 0.43, 3.34.

Sensitivity analysis according to the variables skin color [white/nonwhite; see Supplemental Material, Table S2 (http://dx.doi.org/10.1289/ehp.1103942], diarrhea, parasitic diseases, dehydration, or malnutrition (see Supplemental Material, Table S3), and respiratory diseases (see Supplemental Material, Table S4) seems to support the presented results on the association between pesticide exposure and leukemia among very young children (Table 2).

Adjusted ORs for any exposure to pyrethroid pesticides during pregnancy were 1.80 (95% CI: 1.11, 2.90) for ALL and 3.39 (95% CI: 1.72, 16.78) for AML (Table 3). Adjusted ORs for use of pesticide formulations that included solvents were 1.79 (95% CI: 1.10, 2.92) for ALL and 3.45 (95% CI: 1.76, 6.74) for AML (data not shown).

**Table 3:** Maternal use of pesticides according to chemical class, frequency and type of use during pregnancy; leukemia cases and controls, children < 2 years of age, Brazil, 1999–2007.

Pesticide use during pregnancy	Controls (n = 423) [n (%)]	ALL (n = 193) [n (%)]	AML (n = 59) [n (%)]	ALL vs. controls Crude OR (95% CI)	ALL vs. controls aOR (95% CI)a	AML vs. controls Crude OR (95% CI)	AML vs. controls aORa (95% CI)
Chemical group
None used	316 (74.7)	111 (57.5)	27 (45.7)	1.00	1.00	1.00	1.00
Pyrethroid	89 (21.0)	63 (32.6)	25 (42.4)	2.02 (1.34–3.02)	1.80 (1.11–2.90)	3.29 (1.73–6.20)	3.39 (1.72–16.78)
Organophosphates	7 (1.7)	5 (2.6)	4 (6.8)	2.03 (0.63–6.54)	1.06 (0.26–4.32)	6.69 (1.84–24.29)	5.50 (1.44–21.03)
Other pesticidesb	17 (2.6)	14 (7.3)	3 (5.1)	2.66 (1.30–5.43)	2.96 (1.28–6.84)	2.66 (0.84–8.44)	1.77 (0.35–8.80)
Type of use
None used	316 (74.7)	111 (57.5)	27 (48.2)	1.00	1.00	1.00	1.00
Household	89 (21.0)	62 (32.1)	25 (44.6)	1.98 (1.34–2.93)	1.88 (1.20–2.95)	3.29 (1.82–5.95)	3.12 (1.61–6.05)
Agriculture	7 (1.7)	15 (7.8)	4 (7.1)	6.10 (2.42–15.35)	5.25 (1.83–15.08)	6.69 (1.84–24.29)	7.56 (1.83–31.23)
Frequency
None	315 (79.3)	111 (64.9)	27 (55.1)	1.00	1.00	1.00	1.00
Up to once/week	45 (11.3)	35 (20.5)	13 (26.5)	2.23 (1.38–3.59)	2.19 (1.29–3.69)	3.20 (1.58–6.48)	2.88 (1.35–6.17)
> Once/week	37 (8.7)	25 (13.0)	9 (15.3)	1.82 (1.02–3.25)	2.06 (1.10–3.86)	2.58 (1.09–6.09)	2.94 (1.17–7.38)
Brands with mixed chemicalsc
No	414 (97.9)	183 (94.8)	55 (93.2)	1.00	1.00	1.00	1.00
Yes	9 (2.1)	10 (5.2)	4 (6.8)	2.51 (1.01–6.29)	5.22 (1.44–18.97)	3.35 (0.73–12.35)	6.51 (1.25–33.99)
Distinct pest classes
No	420 (99.3)	186 (96.4)	58 (98.3)	1.00	1.00	1.00	1.00
Yes	3 (0.7)	7 (3.6)	1 (1.7)	5.27 (1.35–20.6)	18.34 (2.01–167.38)	3.35 (1.00–11.23)	8.03 (0.37–174.68)
aaORs by use of oral contraceptives during pregnancy, maternal age and education, child’s birth weight and skin color. bOrganochlorines, cumarines, and others. cExposure to more than one chemical in the same product or distinct products.							

The reported use of pesticide brands containing organophosphates was uncommon (1.7% of control, 2.6% of AML, and 6.8% of ALL mothers). The magnitude of association between such products and AML in very young children was shown as aOR = 5.50 (95% CI: 1.44, 21.03) (Table 3).

Adjusted ORs for pesticide exposure at home were 3.12 (95% CI: 1.61, 6.05) for AML, and 1.88 (95% CI: 1.20, 2.95) for ALL (Table 3). Adjusted ORs for agricultural pesticide exposures were indeed higher: aOR = 5.25; 95% CI: 1.83, 15.08 for ALL, and aOR = 7.56; 95% CI: 1.83, 31.23 for AML.

Compared with no reported pesticide exposure, an aOR of 2.88 (95% CI: 1.35, 6.17) for AML was estimated for the children of women reporting exposure once a week or less, and aOR of 2.94 (95% CI: 1.17, 7.38) for exposure more than once a week (Table 3). For ALL, corresponding aORs were 2.19 (95% CI: 1.29, 3.69) and 2.06 (95% CI: 1.10, 3.86). Adjusted ORs for exposure to commercial products that included multiple pesticides were 5.22 (95% CI: 1.44, 19.0) for ALL, and 6.51 (95% CI: 1.25, 34.0) for AML.

A comprehensive analysis of all individual chemical components included in the reported brands revealed statistically significant associations between leukemia in very young children and maternal exposure to seven pyrethroids and unspecified solvents (Table 4). Estimates of associations with exposure to individual pyrethroids during pregnancy were imprecise due to small numbers of reported exposures. Permethrin, imiprothrin, and esbiothrin exposures during pregnancy were positively associated either with ALL (aOR = 2.47; 95% CI: 1.17, 5.25; aOR = 2.61; 95% CI: 1.06, 6.93; aOR 3.03; 95% CI: 1.13, 8.09, respectively) or AML (aOR = 7.28; 95% CI: 2.60, 20.38; aOR = 3.41; 95% CI: 0.98, 11.90; aOR = 3.19; 95% CI: 0.77, 13.19) at 0–11 months of age.

**Table 4:** Maternal exposure to chemical components of specific pesticides during pregnancy; leukemia cases and controls, children < 2 years of age, Brazil, 1999–2007.

Chemical compound	Controls (n = 423) [n (%)]	ALL (n = 193) [n (%)]	AML (n = 59) [n (%)]	ALL		AML	
				Crude OR (95% CI)	aORa (95% CI)	Crude OR (95% CI)	aORa (95% CI)
Prallethrin
0–11 months
No	234 (55.3)	78 (40.4)	23 (43.4)	1.00	1.00	1.00	1.00
Yes	3 (0.7)	1 (0.5)	2 (3.8)	1.00 (0.10–9.75)	1.52 (0.15–15.32)	6.78 (1.08–42.70)	8.06 (1.17–55.65)
12–23 months
No	155 (36.6)	94 (48.7)	29 (54.7)	1.00	1.00	1.00	1.00
Yes	3 (0.7)	3 (1.5)	0 (0.0)	1.65 (0.33–8.34)	1.16 (0.15–9.12)	—	—
Permethrin
0–11 months
No	211 (49.9)	63 (32.6)	15 (28.3)	1.00	1.00	1.00	1.00
Yes	26 (6.1)	16 (8.3)	10 (18.9)	2.06 (1.04–4.08)	2.47 (1.17–5.25)	5.41 (2.20–13.28)	7.28 (2.60–20.38)
12–23 months
No	128 (30.3)	75 (38.8)	23 (43.4)	1.00	1.00	1.00	1.00
Yes	30 (7.1)	22 (11.4)	6 (11.3)	1.25 (0.67–2.33)	1.47 (0.71–3.08)	1.11 (0.42–2.97)	1.32 (0.43–4.02)
Imiprothrin
0–11 months
No	223 (52.7)	67 (34.7)	20 (37.7)	1.00	1.00	1.00	1.00
Yes	14 (3.3)	12 (6.2)	5 (9.4)	2.85 (1.26–6.46)	2.61 (1.06–6.93)	3.98 (1.30–12.91)	3.41 (0.98–11.90)
12–23 months
No	140 (33.1)	81 (42.0)	22 (41.5)	1.00	1.00	1.00	1.00
Yes	18 (4.3)	16 (8.3)	7 (13.2)	1.54 (0.74–3.18)	1.38 (0.59–3.23)	2.48 (0.93–6.61)	2.85 (0.94–8.62)
Esbiothrin
0–11 months
No	227 (53.7)	68 (35.2)	21 (39.6)	1.00	1.00	1.00	1.00
Yes	10 (2.4)	11 (5.7)	4 (7.5)	3.67 (1.50–9.02)	3.03 (1.13–8.09)	4.32 (1.25–14.98)	3.19 (0.77–13.19)
12–23 months
No	144 (34.0)	83 (43.0)	22 (41.5)	1.00	1.00	1.00	1.00
Yes	14 (3.3)	14 (7.3)	7 (13.2)	1.74 (0.79–3.82)	1.66 (0.67–4.13)	3.27 (1.19–9.00)	3.71 (1.18–11.62)
Tetramethrin
0–11 months
No	214 (50.6)	68 (35.2)	17 (32.1)	1.00	1.00	1.00	1.00
Yes	23 (5.4)	11 (5.7)	8 (15.1)	1.51 (0.70–3.25)	1.56 (0.65–3.72)	4.38 (1.70–11.25)	6.19 (2.07–18.56)
12–23 months
No	134 (31.7)	78 (40.4)	27 (50.9)	1.00	1.00	1.00	1.00
Yes	24 (5.7)	17 (8.8)	2 (3.8)	1.22 (0.66–2.40)	1.35 (0.59–3.07)	0.41 (0.09–1.85)	0.47 (0.09–2.48)
d-Phenothrin
0–11 months
No	234 (55.3)	72 (37.3)	24 (45.3)	1.00	1.00	1.00	1.00
Yes	3 (0.7)	7 (3.6)	1 (1.9)	7.58 (1.91–30.08)	4.16 (0.85–20.29)	3.25 (0.33–32.48)	1.64 (0.16–19.68)
12–23 months
No	155 (36.6)	93 (48.2)	25 (47.2)	1.00	1.00	1.00	1.00
Yes	3 (0.7)	4 (2.1)	4 (7.5)	2.22 (0.49–10.15)	0.69 (0.10–4.88)	8.27 (1.75–39.16)	8.43 (1.59–44.75)
d-Allethrin
0–11 months
No	213 (50.3)	68 (35.2)	17 (32.1)	1.00	1.00	1.00	1.00
Yes	24 (5.7)	11 (5.7)	8 (15.1)	1.43 (0.67–3.08)	1.56 (0.65–3.72)	4.18 (1.63–10.70)	6.19 (2.07–18.56)
12–23 months
No	132 (31.2)	78 (40.4)	27 (50.9)	1.00	1.00	1.00	1.00
Yes	26 (6.1)	19 (9.8)	2 (3.8)	1.24 (0.64–2.38)	1.54 (0.70–3.39)	0.38 (0.08–1.68)	0.46 (0.09–2.40)
Solvents
0–11 months
No	205 (48.5)	59 (30.1)	13 (24.5)	1.00	1.00	1.00	1.00
Yes	32 (7.5)	20 (10.4)	12 (22.6)	2.17 (1.16–4.07)	2.17 (1.06–4.43)	5.91 (2.48–14.10)	6.70 (2.50–17.97)
12–23 months
No	122 (28.8)	70 (36.3)	20 (37.7)	1.00	1.00	1.00	1.00
Yes	36 (8.5)	27 (14.0)	9 (17.0)	1.31 (0.73–2.33)	1.32 (0.66–2.63)	1.52 (0.64–3.64)	1.82 (0.68–4.84)
aaORs by use of oral contraceptives during pregnancy, maternal age and education, child’s birth weight and skin color.								

Among children 11–23 months of age, esbiothrin exposures during pregnancy were positively associated with AML (aOR = 3.71; 95% CI: 1.18, 11.62) (Table 4). Maternal exposure to tetramethrin and d-allethrin was positively associated with AML in children 0–11 months of age (aOR = 6.19; 95% CI: 2.07, 18.56 for both), whereas d-phenothrin was positively associated with AML in children 11–23 months of age (aOR = 8.43; 95% CI: 1.59, 44.75) (Table 4).

## Discussion

Pesticides are complex mixtures that include components such as solvents, humidifying agents, emulsifiers, and additives in addition to active ingredients (Bolognesi 2003; Feron et al. 1998). Furthermore, the seasonal use of distinct formulas for specific purposes makes it difficult to present a qualitative evaluation of exposure to individual substances.

Our findings suggest that children whose mothers were exposed to pesticides 3 months before conception were at least twice as likely to be diagnosed with ALL in the first year of life compared with those whose mothers did not report such exposure. Adjusted ORs for AML in the first year of life ranged from 2.75 (95% CI: 0.96, 7.92) for any pesticide exposure in the first trimester of pregnancy, to 7.04 (95% CI: 2.47, 20.10) for exposure during breastfeeding.

Studies conducted in other countries have also reported positive associations between pesticide exposure and hematopoietic neoplasms in children, especially leukemias and lymphomas (Ma et al. 2002; Meinert at al. 2000; Menegaux et al. 2006; Rudant et al. 2007; Zahm and Ward 1998). A systematic review and meta-analysis of 15 studies of the association between residential exposure to pesticides during selected time windows (preconception, pregnancy, and childhood) and childhood leukemia carried out during 1950–2009 (Turner et al. 2010) reported associations with pregnancy exposure to unspecified pesticides (OR = 1.54; 95% CI: 1.13, 2.11), insecticides (OR = 2.05; 95% CI: 1.80, 2.32), and herbicides (OR = 1.61; 95% CI: 1.20, 2.16). Another meta-analysis of 31 studies of parental occupational exposure to pesticides and childhood leukemia (Wigle et al. 2009) reported associations with occupational exposure to insecticides (OR = 2.72; 95% CI: 1.47, 5.04) and herbicides (OR = 3.62; 95% CI: 1.28, 10.3) during pregnancy.

A French study also examined the association between pesticide exposure and infant leukemia (Rudant et al. 2007). According to use of any pesticide, the observed risk estimates (ORs) were 2.3 (95% CI: 1.9, 2.8) for ALL and 2.2 (95% CI: 1.4, 3.3) for AML. These authors also suggested that a domestic use of pesticides may play a role in the etiology of leukemia, and that prenatal exposure may be a window of fetal vulnerability.

Pesticide exposure during childhood may occur in many ways, either through contamination of their parents’ work clothes or through household residues in water, air, soil, and food (Araújo et al. 2000; Rudant et al. 2007). However, the short latency period for leukemias diagnosed during the first year of life suggests that intrauterine exposures may play a paramount role in this process.

In the Agricultural Health Study—a large prospective cohort study of approximately 49,000 pesticide applicators in the United States—an association between permethrin exposure and multiple myeloma was observed (Rusiecki et al. 2009). Compared with applicators who reported never using permethrin, the risk ratio for multiple myeloma among applicators in the highest tertile of lifetime exposure was 5.72 (95% CI: 2.76, 11.87). Another study of this cohort (Flower et al. 2004) reported a positive association between having a parent who applied pesticides and lymphomas diagnosed among children [age-standardized incidence ratio (SIR) of 2.18 (95% CI: 1.13, 4.19)], but not leukemias in children (SIR = 0.91; 95% CI: 0.47, 1.95).

Moreover, the U.S. Environmental Protection Agency (EPA) and the Canadian Pest Management Regulatory Agency (PMRA) have referred the occurrence of carcinogenicity following permethrin exposure in animal toxicity studies (Weichental et al. 2010). An insecticide containing imiprothrin and deltamethrin that is widely used in Egypt has been evaluated for immunotoxic effects in rats (Emara and Draz 2007). The authors observed that animals exposed to both chemicals, compared with unexposed animals, had altered levels of splenic CD4^+^ and CD8^–^ cells and CD4^+^ and CD8^+^ cells, and concluded that a repeated noncontinous inhalation of imiothropin and deltamethrin causes several immunotoxic effects in other distal sites to the lungs. Other pyrethroids, such as allethrin, cyhalothrin, cypermethrin, deltamethrin, and tetramethrin, have also been suggested to be involved in canine mammary carcinogenesis (Andrade et al. 2010).

This research has some limitations. The hospital-based case–control study design may introduce selection bias depending on the chosen comparison groups (Rudant et al. 2010; Wacholder et al. 1992). We recruited controls with a variety of indications for hospitalization and enrolled controls from general hospitals in the same cities, though not necessarily the same hospitals, in which the cases were diagnosed. On the other hand, the similar origin of cases and controls could theoretically induce the introduction of overmatching in relation to agriculture pesticide exposure. The reports on pesticide exposure in the agricultural set (15 ALL, 4 AML, and 7 controls, Table 3) accounted for 3.3% of all participants. Therefore, we think it improbable that overmatching on agricultural pesticide exposure has distorted the conclusions of this investigation.

Pesticide exposures during the examined time windows were highly correlated, with statistically significant high Pearson’s correlation coefficients, *r* > 0.77. Hence, associations with exposures during specific time of windows could not be accurately determined. Additionally, length of exposure was not evaluated in this study, so associations according to maternal cumulative exposure to pesticides could not be estimated. Finally, sample size was limited, mainly to AML, thus resulting in imprecise estimates of association.

On the other hand, the study has some strengths, being relatively large given that the outcomes are rare. In addition, most previous studies have been based on populations from a limited number of countries, so our study contributes for exploring the role of pesticide exposure during pregnancy and leukemias in children < 2 years of age. Moreover, information on the type of pesticide exposure, time periods of exposure, exposures to individual chemicals (mainly pyrethroids), and data on subgroups of leukemias (ALL, AML), may enhance understanding of the role of maternal exposure to pesticides during pregnancy and leukemia in young children.

## Conclusions

Future research will benefit from exploring the genetic and molecular mechanisms that characterize individual susceptibility to pesticide exposures in the development of leukemia in young children. However, the consistency of our findings with those of similar studies performed in different populations supports recommendations for women of reproductive age to minimize their exposure to pesticides before and during pregnancy and breastfeeding.

## Supplemental Material

(197 KB) PDF
