# Supplementary material for: In Utero Pesticide Exposure and Leukemia in Brazilian Children < 2 Years of Age
Source: Environ Health Perspect. 2012 Oct 22;121(2):269–75. doi: 10.1289/ehp.1103942 (PMC3569673; doi:10.1289/ehp.1103942)
Supplement: (197 KB) PDF [file ehp.1103942.s001.pdf]

Supplemental Material

In Utero Pesticide Exposure and Leukemia in Brazilian Children < 2 Years of Age

Jeniffer Dantas Ferreira, Arnaldo Cézar Couto, Maria do Socorro Pombo-de-Oliveira, Sergio Koifman, and the Brazilian Collaborative Study Group of Infant Acute Leukemia

Table of Contents

Table S1. Geographical distribution of participants, cases and controls, Brazil, 1999-2007 p. 2

Table S2. Associations between maternal pesticide exposure by time of exposure stratified by child's skin color among children < 2 years of age, Brazil, 1999–2007. p. 3

Table S3. Associations between maternal exposure to pesticides by time of exposure among children < 2 years of age (excluding controls with gastrointestinal infections, parasitic diseases, dehydration, malnutrition, or diarrhea), Brazil, 1999–2007. p. 5

Table S4. Associations between maternal exposure to pesticides by time of exposure among children < 2 years of age (excluding controls with tuberculosis, pneumonia, asthma, bronchitis, and bronchiolitis) Brazil, 1999–2007. p. 6

Table S1. Geographical distribution of participants in Brazil, 1999–2007.

State	Cases (*n*)	Controls (*n*)
São Paulo	63	66
Rio de Janeiro	60	129
Bahia	35	68
Minas Gerais	32	42
Rio Grande do Sul	13	23
Distrito Federal and Goiás	12	25
Pernambuco	10	19
Santa Catarina	8	26
Paraná	6	6
Paraíba	5	11
Mato Grosso	4	2
Mato Grosso do Sul	2	2
Rio Grande do Norte	2	2

Table S2. Associations between maternal exposure to pesticides by time of exposure stratified by child's skin color among children < 2 years of age, Brazil, 1999–2007.

**Pesticide exposure/ ethnic group**	**Controls (*n* = 395), *n* (%)**	**ALL (*n* = 193), *n* (%)**	**AML (*n* = 53), *n* (%)**	**ALL**		**AML**	
				**Crude OR (95% CI)**	**Adj OR[a] (95% CI)**	**Crude OR (95% CI)**	**Adj OR[a] (95% CI)**
**Pesticide use**							
**White**							
No	122 (79.7)	75 (57.3)	18 (46.2)	1.00	1.00	1.00	1.00
Yes	31 (20.3)	56 (42.7)	21 (53.8)	2.94 (1.74-4.97)	2.14 (1.21-3.79)	4.59 (2.19-9.65)	4.18 (1.87-9.34)
**Non-white**							
No	184 (71.9)	33 (55.9)	9 (50.0)	1.00	1.00	1.00	1.00
Yes	72 (28.1)	26 (44.1)	9 (50.0)	2.01 (1.13-3.60)	1.97 (1.08-3.60)	2.56 (0.98-6.70)	2.38 (0.87-6.51)
**Periconceptual[b]**							
**White**							
No	136 (88.9)	85 (72.0)	22 (66.7)	1.00	1.00	1.00	1.00
Yes	17 (11.1)	33 (28.0)	11 (33.3)	3.11 (1.63-5.20)	4.42 (1.75-11.13)	4.00 (1.66-9.66)	3.19 (1.63-6.23)
**Non-white**							
No	199 (79.9)	39 (69.6)	10 (58.8)	1.00	1.00	1.00	1.00
Yes	50 (20.1)	17 (30.4)	7 (77.3)	1.74 (0.91-3.32)	1.66 (0.85-3.24)	2.79 (1.01-7.68)	2.43 (0.84-7.03)
**1st Trimester**							
**White**							
No	129 (84.3)	86 (72.9)	24 (72.7)	1.00	1.00	1.00	1.00
Yes	24 (15.7)	32 (27.1)	9 (27.3)	2.00 (1.10-3.63)	1.79 (0.96-3.37)	2.02 (0.84-4.87)	2.14 (0.86-5.34)
**Non-white**							
No	207 (83.5)	39 (69.6)	12 (70.6)	1.00	1.00	1.00	1.00
Yes	41 (16.5)	17 (30.4)	5 (29.4)	2.20 (1.14-4.26)	2.15 (1.08-4.25)	2.01 (0.70-6.29)	1.80 (0.57-5.73)

Table S2 continued

Pesticide exposure/ ethnic group	Controls (*n* = 395), *n* (%)	ALL (*n* = 193), *n* (%)	AML (*n* = 53), *n* (%)	ALL Crude OR (95% CI)	ALL Adj OR[a] (95% CI)	AML Crude OR (95% CI)	AML Adj OR[a] (95% CI)
**2nd Trimester**							
**White**							
No	131(85.6)	90 (72.9)	24 (72.7)	1.00	1.00	1.00	1.00
Yes	22 (14.4)	28 (27.1)	9 (27.3)	1.85 (1.00-3.44)	1.66 (0.86-3.21)	2.23 (0.92-5.43)	2.40 (0.95-6.04)
**Non-white**							
No	205 (82.3)	39 (69.6)	12 (70.6)	1.00	1.00	1.00	1.00
Yes	44 (17.7)	17 (30.4)	5 (29.4)	2.03 (1.05-3.91)	1.92 (0.97-3.78)	1.94 (0.65-5.79)	1.56 (0.49-5.00)
**3rd Trimester**							
**White**							
No	129 (84.3)	91 (77.1)	23 (69.7)	1.00	1.00	1.00	1.00
Yes	24 (15.7)	27 (22.9)	10 (30.3)	1.60 (0.87-2.94)	1.44 (0.75-2.77)	2.34 (0.99-5.53)	2.54 (1.03-6.22)
**Non-white**							
No	201 (81.0)	39 (69.6)	12 (70.6)	1.00	1.00	1.00	1.00
Yes	47 (19.0)	17 (30.4)	5 (29.4)	1.84 (0.97-3.58)	1.77 (0.90-3.49)	1.78 (0.60-5.30)	1.46 (0.46-4.63)
**Breastfeeding[c]**							
**White**							
No	136 (88.9)	92 (78.0)	21 (63.6)	1.00	1.00	1.00	1.00
Yes	17 (11.1)	26 (22.0)	12 (36.4)	2.26 (1.16-4.40)	1.96 (0.97-3.97)	4.57 (1.92-10.91)	5.11 (2.05-12.75)
**Non-white**							
No	210 (84.7)	42 (75.0)	14 (82.4)	1.00	1.00	1.00	1.00
Yes	38 (15.3)	14 (25.0)	3 (17.6)	1.84 (0.92-3.70)	1.81 (0.88-3.71)	1.18 (0.33-4.32)	1.08 (0.28-4.16)

Abbreviations: adj., adjusted; ALL, acute lymphoblastic leukemia; AML, acute myeloid leukemia; OR, odds ratio. [a]Adjusted by oral contraceptive use during pregnancy, maternal age and education, and child's birth weight and skin color. [b]Three months before pregnancy. [c]Three months after delivery.

Table S3. Associations between maternal exposure to pesticides by time of exposure among children < 2 years of age (excluding controls with gastrointestinal infections, parasitic diseases, dehydration, malnutrition, and diarrhea), Brazil, 1999-2007.

Pesticide Exposure	Controls[a] (*n* = 395), *n* (%)	ALL (*n* = 193), *n* (%)	AML (*n* = 53), *n* (%)	ALL		AML	
				Crude OR (95% CI)	Adj OR[b] (95% CI)	Crude OR (95% CI)	Adj OR[b] (95% CI)
**Pesticide use**							
No	295 (74.7)	111 (57.5)	27 (50.1)	1.00	1.0	1.00	1.00
Yes	100 (25.3)	82 (42.5)	32 (49.9)	2.18 (1.51-3.14)	2.12 (1.40-3.21)	3.50 (2.00-6.12)	3.27 (1.76-6.08)
**Periconceptual** [b]							
No	323 (83.7)	127 (71.8)	32 (62.7)	1.00	1.00	1.00	1.00
Yes	63 (16.3)	50 (28.2)	19 (77.3)	2.02 (1.32-3.09)	2.37 (1.49-3.75)	3.04 (1.62-5.71)	3.34 (1.67-6.69)
**1st Trimester**							
No	325 (84.2)	128 (72.3)	36 (70.6)	1.00	1.00	1.00	1.00
Yes	61 (15.8)	49 (27.7)	15 (29.4)	2.04 (1.33-3.13)	2.13 (1.33-3.39)	2.22 (1.15-4.30)	2.07 (1.01-4.24)
**2nd Trimester**							
No	324 (83.7)	132 (74.6)	36 (70.6)	1.00	1.00	1.00	1.00
Yes	63 (16.3)	45 (25.4)	15 (29.4)	1.75 (1.14-2.70)	1.82 (1.14-2.92)	2.14 (1.11-4.15)	1.94 (0.95-3.97)
**3rd Trimester**							
No	318 (82.4)	133 (75.1)	35 (68.6)	1.00	1.00	1.00	1.00
Yes	68 (17.6)	44 (24.9)	16 (31.4)	1.55 (1.01-2.38)	1.67 (1.05-2.66)	2.14 (1.13-4.08)	2.02 (1.00-4.08)
**Breastfeeding**[c]							
No	333 (86.5)	137 (77.4)	35 (68.6)	1.00	1.00	1.00	1.00
Yes	52 (13.5)	40 (22.6)	16 (31.4)	1.87 (1.18-2.96)	1.98 (1.21-3.26)	2.93 (1.51-5.66)	2.90 (1.41-5.95)

Abbreviations: adj., adjusted; ALL, acute lymphoblastic leukemia; AML, acute myeloid leukemia; OR, odds ratio.
[a]Adjusted by oral contraceptive use during pregnancy, maternal age and education, and child's birth weight and skin color [b]Three months before pregnancy. [c]Three months after delivery.

Table S4. Associations between maternal exposure to pesticides by time of exposureamong children < 2 years of age (excluding controls with tuberculosis, pneumonia, asthma, bronchitis, and bronchiolitis)Brazil, 1999–2007.

Pesticide Exposure	Controls[a] (*n* = 283), *n* (%)	ALL (*n* = 193), *n* (%)	AML (*n* = 53), *n* (%)	ALL		AML	
				Crude OR (95% CI)	Adj OR[b] (95% CI)	Crude OR (95% CI)	Adj OR[b] (95% CI)
**Pesticide use**							
No	209 (73.9)	111 (57.5)	27 (50.1)	1.00	1.00	1.00	1.00
Yes	74 (26.1)	82 (42.5)	32 (49.9)	2.10 (1.43-3.07)	2.02 (1.30-3.14)	3.67 (1.90-5.96)	3.21 (1.68-6.12)
**Periconceptual[b]**							
No	228 (83.2)	127 (71.8)	32 (62.7)	1.00	1.00	1.00	1.00
Yes	46 (16.8)	50 (28.2)	19 (77.3)	1.93 (1.24-3.01)	2.19 (1.35-3.58)	2.99 (1.53-5.53)	3.04 (1.48-6.23)
**1st Trimester**							
No	230 (83.6)	128 (72.3)	36 (70.6)	1.00	1.00	1.00	1.00
Yes	45 (16.4)	49 (27.7)	15 (29.4)	1.88 (1.21-2.94)	1.96 (1.20-3.21)	2.05 (1.05-4.02)	1.89 (0.90-3.97)
**2nd Trimester**							
No	226 (82.2)	132 (74.6)	36 (70.6)	1.00	1.00	1.00	1.00
Yes	49 (17.8)	45 (25.4)	15 (29.4)	1.60 (1.02-2.51)	1.62 (0.99-2.66)	1.96 (1.00-3.83)	1.69 (0.81-3.54)
**3rd Trimester**							
No	222 (80.7)	133 (75.1)	35 (68.6)	1.00	1.00	1.00	1.00
Yes	53 (19.3)	44 (24.9)	16 (31.4)	1.42 (0.91-2.22)	1.50 (0.91-2.44)	1.97 (1.02-3.79)	1.18 (0.88-3.75)
**Breastfeeding[c]**							
No	234 (85.4)	137 (77.4)	35 (68.6)	1.00	1.00	1.00	1.00
Yes	40 (14.6)	40 (22.6)	16 (31.4)	1.75 (1.09-2.83)	1.78 (1.05-3.01)	2.74 (1.40-5.38)	2.52 (1.20-5.29)

Abbreviations: adj., adjusted; ALL, acute lymphoblastic leukemia; AML, acute myeloid leukemia; OR, odds ratio.
[a]Adjusted by oral contraceptive use during pregnancy, maternal age and education, and child's birth weight and skin color. [b]Three months before pregnancy. [c]Three months after delivery.
